# Estriol Is Likely Not Associated With Mouth Ulcerations in Pharmacovigilance Data

**DOI:** 10.1016/j.identj.2026.109509

**Published:** 2026-03-27

**Authors:** Ruwen Böhm, Thomas Herdegen

**Affiliations:** Department of Radiation Oncology, Universitätsklinikum Schleswig-Holstein, Kiel, Germany; Institute of Experimental and Clinical Pharmacology, Universitätsklinikum Schleswig-Holstein, Kiel, Germany

To the Editor,

During medical literature monitoring (MLM) for the drug estriol we were alerted to the publication by Zhang et al. Their pharmacovigilance study proposes that estriol is statistically significantly associated with “oral mucosal ulcerations” in US pharmacovigilance data.^1^ The supplemental material further specifies a total of 25 purportedly individual safety reports (ISRs) for estriol and at least one of the MedDRA terms used as an indication of “oral mucosal ulcerations”, among them the MedDRA preferred term (PT) “mouth ulceration”. The authors do not comment further on this finding. This extremely high number of ISRs delivers a strong statistical signal, prompting further exploration of the ISRs, interpretation of the signal, and research of a plausible pharmacological mechanism of action.

Estrogens exert a protective function on mucosal tissue, and there are longstanding, albeit limited data that both primary estrogens, estradiol and estriol, can alleviate oral mucosal damage – besides the well-known protection of vaginal mucosa - due to postmenopause or ovarian dysfunction.^2^, ^3^, ^4^, ^5^ Estradiol is not associated with mouth ulcerations in a multivariable Mendelian randomization analysis of biobank data.^6^

Surprisingly, only estriol but not estradiol, the much more pharmacodynamically effective estrogen, was associated with damage of oral mucosa according to Zhang et al. Inspection of ISRs with the drug “estriol” and the PT “mouth ulceration” yields 34 cases (openFDA data last updated 2025-10-30, analysed with OpenVigilFDA^7^). For an adverse effect on oral mucosa, estrogens need to be applied orally or reach systemic exposure with effective plasma concentrations after some other route of application. Estriol was always used topically for vulvovaginal disorders in the majority of these cases, so a systemic effect on the oral mucosa is extremely unlikely. Topical application of estradiol such as vaginal gel acts locally but does not affect the circulating levels of estradiol.^8^

Most of these ISRs are multiplications of two original cases: a 56-year-old female with 6 drugs and an 86-year-old female with varying lists of more than 40 drugs. These multiplications substantially contributed to the disproportionality and thus the strong pharmacovigilance signal. The authors had been contacted but declined to comment on this issue, so for the time being, faulty data cleaning appears to be the best explanation for this signal.

Further inspection of the results of Zhang et al. reveals a mixture of drug substance and drug product names in the results, indicating missing or faulty drug name mapping.

This reevaluation of the results has important implications beyond this single detected but invalid signal: (1) The regulatory requirement for individual marketing authorisation holders to submit ISR for all cases that they became aware of (eg, through MLM), even if they do not belong to their own marketed medicinal product, has a high risk of multiplication of single cases. Subsequent pharmacovigilance analyses like those by Zhang et al. will further foster these signals and might in turn influence MLM of other companies. This leads to self-propagating and self-amplifying pharmacovigilance signals. Nowadays, AI-driven research tools are fed by these data and will assign the ever repeating signal a higher importance and confidence. The tools such as Perplexity or Elicit will learn these findings and further repeat them. (ii) Today’s high-throughput life science technology and computing power allow screening of vast datasets. Obviously, the screening methods are optimized for high sensitivity. E.g., there is no correction for multiple testing established in pharmacovigilance research. This and other underlying methodological weaknesses of pharmacovigilance data quality result in a rather low specificity. It is thus imperative to check and curate any signals found.^9^,^10^ Zhang et al. did not perform deep analyses of their signals, which is understandable given the high number of results.

In conclusion, the reported association between estriol and oral mucosal ulcerations appears to be driven primarily by duplicated safety reports and limitations in drug-name mapping rather than by a plausible pharmacological mechanism. We therefore encourage authors to implement robust case-level deduplication, standardized drug coding, and clinical plausibility checks before drawing conclusions from statistical signals. More broadly, regulators, marketing authorisation holders, and database curators should consider measures to reduce artificial signal amplification arising from mandatory ISR submissions and repeated case propagation.
